# Silica-Supported Polyphosphoric Acid in the Synthesis of 4-Substituted Tetrahydroisoquinoline Derivatives

**DOI:** 10.3390/molecules18021869

**Published:** 2013-02-01

**Authors:** Stanimir Manolov, Stoyanka Nikolova, Iliyan Ivanov

**Affiliations:** Department of Organic Chemistry, University of Plovdiv, 24 Tzar Assen str., 4000 Plovdiv, Bulgaria

**Keywords:** 4-substituted tetrahydroisoquinoline, cherylline analogues, α-amidoalkylation reaction, silica-supported polyphosphoric acid (SiO_2_-PPA)

## Abstract

We report herein an application of an α-amidoalkylation reaction, as an alternative efficient synthesis of 4-aryl- and 4-methyl-1,2,3,4-tetrahydroisoquinoline derivatives. The amides required for this purpose would result from reaction of aminoacetaldehyde dimethylacetal with different substituted benzenes in polyphosphoric acid, followed by acylation of the obtained amines with different acid chlorides or sulfochlorides. We compared the cyclisation step using conventional (milieu of acetic-trifluoracetic acid = 4:1) and solid supported reagents (SiO_2_/PPA), as recovered, regenerated and reused without loss of its activity catalyst. We found that in comparison to conventional methods, the yields of the reaction are greater and the reaction time is shorter.

## 1. Introduction

Very rare natural 4-aryl-1,2,3,4-tetrahydroisoquinoline alkaloids have been isolated from *Crinum powellii* var. alba and other *Crinum* species [1]. Due to the uniqueness of the structure and potential medicinal properties of the 4-arylisoquinoline derivatives [2,3,4,5], many synthetic routes for these compounds [6,7,8,9,10,11,12,13,14,15] and especially cherylline have been reported. The Amaryllidaceae [6,7,8,9,10,11,12,13,14,15,16] have been one of the most studied families of plants because of their alkaloids composition. In 1970, Brossi *et al.* [1] isolated from *Crinum powelli* an alkaloid with a 4-aryl-1,2,3,4-tetrahydroisoquinoline structure named cherylline. Some of them are valuable medicines with centrally stimulating, thymoleptic, and antiarrhythmic actions [2,17]. Others are calcium antagonistic [18], and exhibit broad spectrum of activity such as antibacterial [19], antiplasmodial [3,4,16,20,21], estrogen agonist/antagonist activity [22], and serotin (5-HT) re-uptake [23].

The importance of these heterocyclic compounds has generated a considerable number of synthetic approaches. Hence, the formation of the nitrogen-containing ring of 4-substituted 1,2,3,4-tetrahydroisoquinolines has been achieved with several reactions such as Bischler-Napieralski [24,25,26], intramolecular Horner [27], Friedel-Crafts [21,27,28,29,30,31,32] Pictet-Spengler [33], and oxazoline-driven chemistry [34], among others [25,35,36,37,38,39,40,41,42]. Most of the reported methods for the synthesis of these compounds are either multistep or low yielding. 

In our previous investigations we report application of polyphosphoric acid (PPA) as a cyclisation agent for the construction of tetrahydroisoquinoline ring systems. Polyphosphoric acid is a strong mineral acid which has powerful dehydrating properties and is widely used for intramolecular and intermolecular acylations, heterocyclic synthesis, and acid-catalyzed reactions. For instance, in recent reports the cyclization reactions of *N*,*N*'-bis(oxotrifluoroalkenyl)-1,3-phenylenediamines in PPA medium [43], the acylation of benzene and its derivatives with 2-, 3-, 4-aminobenzoic and 4-aminophenylacetic acid in PPA to aminobenzophenones [44], and the reaction of tryptamine with carboxylic acid in PPA to afford 1-substitued-3,4-dihydro-9*H*-β-carboline derivatives [45], *etc*, were described. However, the use of PPA has several drawbacks: since 10- to 50-fold excess is generally employed, it is difficult to pour and stir at room temperature, and it is necessary to carefully neutralize the reaction mixtures before the product extraction.

Recently, PPA/SiO_2_ has been used as an efficient heterogeneous catalyst for many organic transformations. PPA/SiO_2_ has some advantages including its low cost, ease of preparation, and ease of handling. In addition, the catalyst can be easily separated from the reaction mixtures by simple filtration and is reusable. Previously [46], the conversion of carbonyl compounds into oxathioacetals or dithioacetals using PPA/SiO_2_ and a convenient method for the synthesis of isoxazole derivatives using PPA/SiO_2_ as a reusable catalyst have been reported [47]. In the last several years the development of non-toxic, low cost, eco-friendly, recycable catalyst systems which give high productivity under mild reaction conditions has received much attention in organic synthesis [48]. Solid supported catalysts [49,50] have gained much prominence due to their inherent economic and environmental benefits, ease of handling, easy catalyst separation and regeneration, thermal stability and long catalytic life [51]. Since the activity and selectivity of a reagent dispersed on the surface of the support is improved as the effective surface area of reagent can be increased manifold, they are expected to perform better than the individual reagents [52]. Low toxicity, moisture, air tolerance and low price are other common features that make the use of solid supported reagents attractive alternative to the conventional catalysts.

## 2. Results and Discussion

**Scheme 1:**
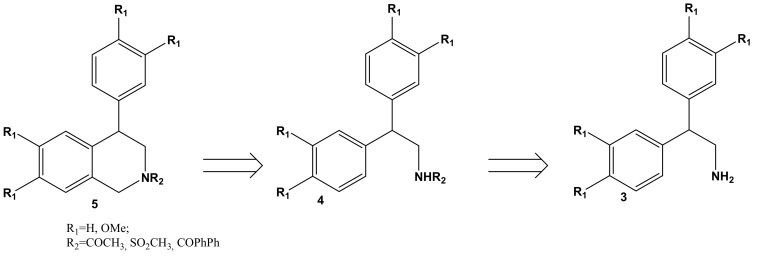
Retrosynthetic scheme for the synthesis of 4-substituted tetrahydroisoquinolines.

**Scheme 2:**
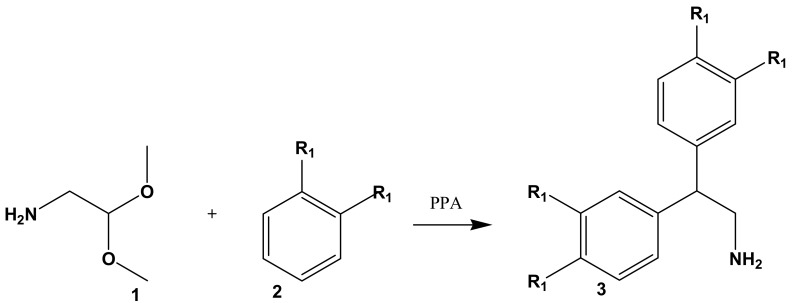
Synthesis of amines **3**.

Our retrosynthetic analysis of 4-substituted 1,2,3,4-tetrahydroisoquinolines is depicted in Scheme 1. We anticipated that **5** could be constructed from amides **4** via an α-amidoalkylation reaction. The required amides **4** can be prepared from the amines **3** via acylation with different acid chlorides. The amines **3** would arise from reaction [53] of aminoacetaldehyde dimethylacetal (**1**) with benzene or substituted benzenes **2** (Scheme 2).

For the synthesis of the required starting materials Klumpp’s [53] protocol using amino acetals and benzene in TfOH to give the corresponding products with good yields can be used. We carried out the same reaction in polyphosphoric acid instead of TfOH. We found that aminoacetaldehyde dimethylacetal reacts with excess of 1,2-dimethoxybenzene (veratrol) in PPA at rt for 5 to 10 min to give a good (82%) yield of the corresponding product **3**. The same product with unsubstituted benzene was obtained after 30 min at 80 °C with 42% yield (Scheme 2, Table 1).

**Scheme 3:**
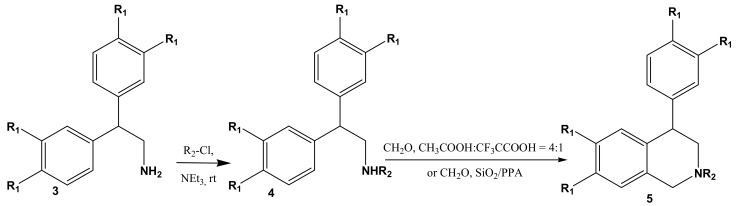
Synthesis of 4-aryl tetrahydroisoquinolines.

The amides **4** required for the next step of our synthesis, were prepared by acylation of the amines **3** with different acid chlorides or sulfochlorides. The next step was cyclization of the newly synthesized amides (Scheme 3, Table 2).

We compared the cyclisation step using either mixture of acetic-trifluoracetic acid = 4:1 or silica-supported PPA (SiO_2_-PPA). We found that with SiO_2_-PPA the reaction yields are greater and the reaction times are shorter (Table 3). The shortest time and best yield were achieved at 80 °C, using 0.06 g of catalyst and 1 h reflux. As described earlier [54] the catalyst was recovered quantitatively and used in the reaction three times. The recovered catalyst did not show any significant loss of activity.

**Scheme 4:**
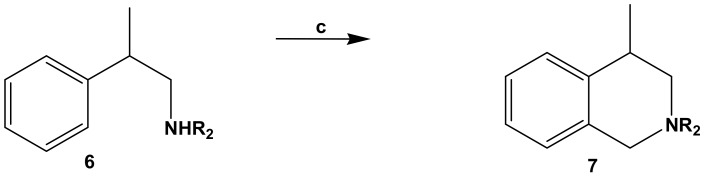
Synthesis of 4-methyl tetrahydroisoquinolines.

The same protocol was applied for the synthesis of 4-methyl-1,2,3,4-tetrahydroisoquinoline derivatives which are interesting as potential anaesthetics and antispasmodics [55] (Scheme 4, Table 4).

## 3. Experimental 

### 3.1. General

Reagents and chemicals were purchased from commercial sources (Sigma-Aldrich S.A. and Riedel-de Haën) and used as received. Melting points were determined on a Boetius hot stage apparatus and are uncorrected. Spectra were recorded on a Bruker Avance DRX250 spectrometer (BAS-IOCCP, Sofia). ^1^H-NMR and ^13^C-NMR spectra were taken in CDCl_3_ (unless otherwise specified) at 250 MHz and 62.5 MHz respectively. Chemical shifts were given in part per million (ppm) relative and were referenced to TMS (δ = 0.00 ppm) as an internal standard and coupling constants are indicated in Hz. All the NMR spectra were taken at rt (ac. 295 K). Elemental analyses were performed with a vario EL III. TLC was carried out on precoated 0.2 mm Fluka silica gel 60 plates, using diethyl ether/*n*-hexane = 1:1 as chromatographic system. Merck silica gel 60 (0.063–0.2 mm) was used for column chromatographic separation. Polyphosphoric acid was obtained from 85% phosphoric acid and P_2_O_5_ (1:1 w/w).

### 3.2. Preparation of PPA/SiO_2_ Catalyst General Procedure

PPA (4.0 g) was charged in the round-bottom flask, and CHCl_3_ (100 mL) was added. After the mixture was stirred at 50 °C for 1 h, followed by SiO_2_ (16.0 g, 70–230 mesh) was added to the solution, and the mixture was stirred for another 1 h. CHCl_3_ was removed by evaporation, and the resulting solid was dried in vacuo at room temperature for 3 h. Used PPA/SiO_2_ was regenerated as follows: PPA/SiO_2_ was recovered by filtration from the reaction mixture, and then it was put in the 50 mL round-bottom flask and dried in vacuum at 100 °C for 2 h.

### 3.3. Typical Procedure for the Synthesis of 2-Amino-1,1-diphenylethanes **3**

Aminoacetaldehyde dimethylacetal (**1**, 0,210 g, 2 mmol) was dissolved in the corresponding benzene (22.47 mmol) in an open flask and polyphosphoric acid (3 g) was added. The reaction mixture was stirred for 20 min at rt. After completion of the reaction, the mixture was poured over ice. The organic layer was removed and after that the water layer the solution was neutralized with sodium hydroxide, then extracted with CH_2_Cl_2_ (3 × 20 mL). Combined extracts were dried (Na_2_SO_4_) and concentrated. The products, after evaporation of the solvent, were purified by column chromatography on silica gel using Et_2_O as eluent.

### 3.4. 2,2-Diphenylethanamine (**3a**): Known Compound [53]

*2,2-Bis(3,4-dimethoxyphenyl)ethanamine* (**3b**). ^1^H-NMR: 1.97 (broad s, 2H, NH_2_), 3.29 (d, 2H, *J* = 7.6 Hz), 3.85 (s, 6H), 3.87 (s, 6H), 3.93 (ddd, 1H, *J* = 4.0, 6.0, 10.0 Hz), 6.76–6.86 (m, 6H); ^13^C-NMR: 149.0, 147.7, 135.3, 119.7, 111.6, 111.3, 55.9, 53.8, 47.1; Anal. calc. for C_18_H_23_NO_4_: C, 68.12; H, 7.30; N, 4.41; found C, 68.18; H, 7.21; N, 4.38.

### 3.5. Acylation of Amines **3**: Typical Procedure for the Synthesis of Amides **4** and **6**

To solution of amine **3** (1 mmol) in dichloromethane (15 mL) equal amount of acetyl chloride, methanesulfonyl chloride or biphenyl-4-carbonyl chloride was added. After 10 min a little excess of triethylamine was added. After 30 min the solution was washed with diluted hydrochloric acid, saturated solution of Na_2_CO_3_ and water. The organic layer was dried (Na_2_SO_4_), concentrated and filtered on short column with neutral Al_2_O_3_.

*N-(2,2-Diphenylethyl)acetamide* (**4a**). ^1^H-NMR: 1.87 (s, 3H), 3.88 (dd, 2H, *J* = 5.9, 7.9 Hz), 4.17 (t, 1H, *J* = 8.0 Hz), 5.46 (s, 1H, NH), 7.22–7.25 (m, 6H), 7.28–7.31 (m, 4H); ^13^C-NMR: 170.1, 141.9, 128.8, 128.1, 126.9, 50.6, 43.9, 23.3; Anal. calc. for C_16_H_17_NO: C, 80.30; H, 7.16; N, 5.85; found C, 80.26; H, 7.23; N, 5.78.

*N-(2,2-Diphenylethyl)methanesulfonamide* (**4b**). ^1^H-NMR: 2.81 (s, 3H), 3.76 (d, 2H, *J* = 7.9 Hz), 4.20 (t, 1H, *J* = 8.0 Hz), 7.20–7.28 (m, 6H), 7.29–7.36 (m, 4H); ^13^C-NMR: 140.9, 129.0, 128.0, 127.3, 51.4, 47.6, 40.5; Anal. calc. for C_15_H_17_NO_2_S: C, 65.43; H, 6.22; N, 5.09; S, 11.64; found C, 65.37; H, 6.29; N, 5.01; S, 11,71.

*N-(2,2-Diphenylethyl)-[1,1'-biphenyl]-4-carboxamide* (**4c**). ^1^H-NMR (CF_3_COOD): 4.36 (d, 2H, *J* = 7.8 Hz), 4.48 (dd, 1H, *J* = 7.0, 9.4 Hz), 7.26–7.36 (m, 3H), 7.35 (s, 5H), 7.38–7.49 (m, 5H), 7.55–7.72 (m, 6H); ^13^C-NMR: 148.4, 139.9, 138.4, 128.7, 128.6, 128.5, 127.7, 127.5, 127.4, 127.2, 126.6, 121.0, 112.0, 107.5, 49.8, 46.5; Anal. calc. for C_27_H_23_NO: C 85.91; H, 6.14; N, 3.71; found C 85.97; H, 6.08; N, 3.78.

*N-(2,2-Bis(3,4-dimethoxyphenyl)ethyl)acetamide* (**4d**). ^1^H-NMR: 1.91 (s, 3H), 3.82–3.90 (m, 2H), overlapped with 3.85 (s, 6H), 3.87 (s, 6H), 4.08 (t, 1H, *J* = 7.9 Hz), 5.46 (t, 1H, *J* = 5.7 Hz, NH), 6.76 (dd, 2H, *J* = 1.9, 5.8 Hz), 6.81 (d, 2H, *J* = 1.9 Hz), 6.83 (s, 1H), 6.86 (s, 1H); ^13^C-NMR: 170.0, 149.1, 147.9, 134.5, 119.7, 111.4, 111.3, 55.9, 55.8, 49.6, 44.0, 23.3; Anal. calc. for C_20_H_25_NO_5_: C, 66.83; H, 7.01; N, 3.90; found C, 66.75; H, 7.09; N, 3.86.

*N-(2,2-Bis(3,4-dimethoxyphenyl)ethyl)methanesulfonamide* (**4e**). ^1^H-NMR: 2.84 (s, 3H), 3.67 (d, 2H, *J* = 7.7 Hz), 3.83 (s, 3H), 3.85 (s, 3H), 4.09 (t, 1H, *J* = 7.8 Hz), 4.32 (broad s, 1H, NH), 6.74 (dd, 2H, *J* = 1.8, 9.8 Hz), 6.80 (d, 2H, *J* = 1.9 Hz), 6.83 (d, 2H, *J* = 8.2 Hz); ^13^C-NMR: 149.2, 148.2, 133.6, 119.6, 111.5, 111.4, 56.0, 55.9, 50.4, 47.7, 40.4; Anal. calc. for C_19_H_25_NO_6_S: C, 57.70; H, 6.37; N, 3.54; S, 8.11; found C, 57.78; H, 6.32; N, 3.61; S, 8.01.

*N-(2,2-Bis(3,4-dimethoxyphenyl)ethyl)-[1,1'-biphenyl]-4-carboxamide* (**4f**). ^1^H-NMR: 3.82 (s, 6H), 3.86 (s, 6H), 4.05 (dd, 2H, *J* = 5.7, 7.7 Hz), 4.24 (t, 1H, *J* = 7.9 Hz), 6.20 (t, 1H, *J* = 5.5 Hz, NH), 6.82 (dd,6H, *J* = 1.1, 10.9 Hz), 7.36–7.47 (m, 3H), 7.54–7.70 (m, 6H); ^13^C-NMR: 167.2, 149.2, 148.1, 144.3, 139.9, 134.6, 133.2, 128.9, 128.0, 127.3, 120.0, 111.6, 111.4, 55.9, 50.0, 44.6; Anal. calc. for C_31_H_31_NO_5_: C, 74.83; H, 6.28; N, 2.81; found C, 74.75; H, 6.34; N, 2.79.

*N-(2-Phenylpropyl)acetamide* (**6a**). ^1^H-NMR: 1.20 (d, 3H, *J* = 7.0 Hz), 1.87 (s, 3H), 2.86 (ddd, 1H, *J* = 2.2, 7.0, 13.2 Hz), 3.14 (ddd, 1H, *J* = 4.8, 8.8, 13.4 Hz), 3.56 (ddd, 1H, *J* = 6.1, 7.0, 13.1 Hz), 5.30 (broad s, 1H, NH), 7.14 (ddd, 3H, *J* = 1.8, 4.3, 5.5 Hz), 7.25 (dt, 2H, *J* = 2.0, 3.5 Hz); ^13^C-NMR: 170.0, 144.1, 128.8, 127.2, 126.8, 46.2, 39.7, 23.3, 19.5; Anal. calc. for C_11_H_15_NO: C, 74.54; H, 8.53; N, 7.90; found C, 74.48; H, 8.60; N, 7.85.

*N-(2-Phenylpropyl)methanesulfonamide* (**6b**). ^1^H-NMR: 1.24 (d, 3H, *J* = 7.0 Hz), 2.69 (s, 3H), 2.89 (dd, 1H, *J* = 6.7, 14.7 Hz), 3.11–3.19 (m, 1H), 3.21–3.33 (m, 1H), 4.22 (t, 1H, *J* = 6.8 Hz, NH), 7.13–7.21 (m, 3H), 7.23–7.30 (m, 2H); ^13^C-NMR: 143.1, 128.9, 127.3, 127.2, 50.0, 40.4, 40.3, 19.0; Anal. calc. for C_10_H_15_NO_2_S: C, 56.31; H, 7.09; N, 6.57; S, 15.03; found C, 56.26; H, 7.15; N, 6.49; S, 15.09.

*N-(2,2-Bis(3,4-dimethoxyphenyl)ethyl)-[1,1'-biphenyl]-4-carboxamide* (**6c**). ^1^H-NMR: 1.28 (d, 3H, *J* = 7.0 Hz), 2.95–3.09 (m, 1H), 3.35 (ddd, 1H, *J* = 4.9, 8.7, 13.5 Hz), 3.78 (ddd, 1H, *J* = 6.1, 6.9, 13.2 Hz), 6.00 (t, 1H, *J* = 5.8 Hz, NH), 7.15–7.20 (m, 3H), 7.25–7.32 (m, 3H), 7.33–7.39 (m, 2H), 7.59–7.63 (m, 2H); ^13^C-NMR: 167.2, 144.2, 144.1, 140.0, 133.4, 128.93, 128.85, 128.0, 127.34, 127.29, 127.2, 127.0, 46.7, 39.9, 19.3; Anal. calc. for C_31_H_31_NO_5_: C, 74.83; H, 6.28; N, 2.81; found C, 74.79; H, 6.33; N, 2.78.

### 3.6. Cyclization of Amides **4** and **6** in Acetic/Trifluoracetic Acid Milieu: Typical Procedure

2-Phenylethylamides (3 mmol) and paraformaldehyde (5 mmol) were dissolved in a mixture of CH_3_COOH/CF_3_COOH = 4:1 (5 mL) at 80–100 °C for 1 h. The solution was poured on the crushed ice and extracted with CH_2_Cl_2_ (3 × 20 mL). The extract was washed with 20% aq.Na_2_CO_3_ (2 × 30 mL) and dried (Na_2_SO_4_). The solvent was distilled and the products were purified by recrystallization (Et_2_O).

### 3.7. Cyclization of Amides **4** and **6** in SiO_2_-Supported Milieu: Typical Procedure

2-Phenylethylamides (3 mmol) and paraformaldehyde (5 mmol) were dissolved in a C_2_H_4_Cl_2_ (10 mL) at 80 °C, 0.06 g of catalyst (SiO_2_/PPA) and 1h reflux. After the completion of the reaction the reaction mixture was cooled and the catalyst was separated by simple filtration. 

*1-(4-Phenyl-3,4-dihydroisoquinolin-2(1H)-yl)ethanone* (**5a**). ^1^H-NMR: 1.67 (s, 3H), 3.82 (d, 2H, *J* = 4.4 Hz), 4.23 (t, 1H, *J* = 4.2 Hz), 4.52 (d, 1H, *J* = 17.1 Hz), 5.23 (d, 1H, *J* = 17.4 Hz), 6.96–7.06 (m, 3H), 7.22–7.29 (m, 6H); ^13^C-NMR: 169.9, 142.7, 136.0, 133.6, 129.5, 128.7, 128.5, 127.1, 126.6, 51.2, 45.4, 44.3, 20.8; Anal. calc. for C17H17NO: C, 81.24; H, 6.82; N, 5.57; O, 6.37; found C, 81.28; H, 6.77; N, 5.60.

*2-(Methylsulfonyl)-4-phenyl-1,2,3,4-tetrahydroisoquinoline* (**5b**). ^1^H-NMR: 3.47 (dd, 1H, *J* = 7.4, 12.5 Hz), 3.86 (ddd, 1H, *J* = 1.0, 5.0, 12.4 Hz), 4.33 (t, 1H, *J* = 6.3 Hz), 4.57 (q, 2H, *J* = 15.4, 15.38 Hz), 6.94–6.98 (m, 1H), 7.11–7.18 (m, 4H), 7.21–7.31 (m, 4H); ^13^C-NMR: 142.5, 136.2, 132.2, 129.9, 128.9, 128.7, 127.2, 127.0, 126.3, 50.9, 47.6, 45.1, 36.4; Anal. calc. for C_16_H_17_NO_2_S: C, 66.87; H, 5.96; N, 4.87; S, 11.16; found C, 66.91; H, 5.91; N, 4.90; S, 11.11.

*Biphenyl-4-yl(4-phenyl-3,4-dihydroisoquinolin-2(1H)-yl)methanone* (**5c**). ^1^H-NMR (CF_3_COOD): 4.36 (d, 2H, *J* = 7.8 Hz), 4.48 (dd, 1H, *J* = 7.0, 9.4 Hz), 7.26–7.36 (m, 3H), 7.35 (s, 5H), 7.38–7.49 (m, 5H), 7.55–7.72 (m, 6H); ^13^C-NMR: 148.4, 139.9, 138.4, 128.7, 128.6, 128.5, 127.7, 127.5, 127.4, 127.2, 126.6, 121.0, 112.0, 107.5, 49.8, 46.5; Anal. calc. for C_28_H_23_NO C, 86.34; H, 5.95; N, 3.60; found C, 86.39; H, 5.89; N, 3.58.

*1-(4-(3,4-Dimethoxyphenyl)-6,7-dimethoxy-3,4-dihydroisoquinolin-2(1H)-yl)ethanone* (**5d**). ^1^H-NMR (CDCl_3_): 1.66 (s, 3H), 3.74 (s, 3H), 3.81 (s, 3H), 3.87 (s, 3H), 3.90 (s, 3H), 4.90 (t, 1H, *J* = 0.96 Hz), 4.93 (d, 2H, *J* = 1.09 Hz), 5.34–5.35 (m, 2H), 6.47 (s, 1H), 6.53 (s, 1H), 6.68–6.69 (m, 2H), 6.82 (s, 1H); ^13^C-NMR (CDCl_3_): 169.9, 149.0, 148.3, 148.1, 148.0, 147.9, 135.5, 120.7, 111.8, 111.4, 111.2, 109.2, 108.6, 56.2, 56.0, 55.93, 55.89, 51.5, 44.6, 43.9, 20.9. Anal. calc. for C_21_H_25_NO_5_: C, 67.91; H, 6.78; N, 3.77; found C, 67.96; H, 6.74; N, 3.82.

*4-(3,4-Dimethoxyphenyl)-6,7-dimethoxy-2-(methylsulfonyl)-1,2,3,4-tetrahydroisoquinoline* (**5e**). ^1^H-NMR (CDCl_3_): 2.72 (s, 3H), 3.41 (dd, 1H, *J* = 7.42, 12.37 Hz), 3.72 (s, 3H), 3.81–3.86 (m, 1H), overlapped with 3.84 (s, 3H), 3.89 (s, 3H), 3.90 (s, 3H), 4.21 (dd, 1H, *J* = 5.58, 6.24 Hz), 4.51 (q, 2H, *J* = 6.25, 15.0 Hz), 6.46 (s, 1H), 6.64 (s, 1H), 6.67–6.71 (m, 2H), 6.81–6.85 (m, 1H); ^13^C-NMR (CDCl_3_): 149.0, 148.2, 148.1, 135.0, 128.2, 124.1, 121.0, 112.1, 111.9, 111.2, 108.5, 56.0, 55.9, 51.1, 47.3, 44.3, 36.3. Anal. calc. for C_20_H_25_NO_6_S: C, 58.95; H, 6.18; N, 3.44; S, 7.87; found C, 58.99; H, 6.14; N, 3.45; S, 7.81.

*Biphenyl-4-yl[4-(3,4-dimethoxyphenyl)-6,7-dimethoxy-3,4-dihydroisoquinolin-2(1H)-yl]methanone* (**5f**). ^1^H-NMR: 3.73 (s, 3H), 3.76 (s, 3H), 3.91 (s, 3H), 3.94 (s, 3H), 4.09 (s, 1H), 4.65 (d, 1H, *J* = 17.0 Hz), 5.34 (d, 1H, *J* = 16.6 Hz), 6.51 (s, 2H), 6.81 (d, 2H, *J* = 7.7 Hz), 6.92 (d, 2H, *J* = 6.8 Hz), 7.36–7.50 (m, 6H), 7.57–7.65 (m, 4H); ^13^C-NMR: 171.3, 149.2, 148.5, 148.14, 148.07, 142.2, 140.2, 135.2, 134.5, 128.9, 127.5, 127.4, 127.1, 126.8, 125.3, 112.0, 111.7, 111.2, 108.8, 56.03, 55.99, 55.94, 55.87, 52.2, 51.8, 44.6. Anal. calc. for C_32_H_31_NO_5_: C, 75.42; H, 6.13; N, 2.75; found C, 75.50; H, 6.10; N, 2.81. 

## 4. Conclusions 

In conclusion, we have developed a highly efficient SiO_2_-PPA catalyzed method for the construction of 4-aryl- or 4-methyl-1,2,3,4-tetrahydroisoquinoline ring systems, as analogues of biologically-active compounds. The catalyst is completely recoverable and the efficiency of the catalyst remains unaltered even after three to four cycles. It is also noticed that the cyclisation using PPA–SiO_2_ proceeds rapidly and is superior to the reported procedures with respect to yield and amount of the catalyst employed. 

## Figures and Tables

*Reagents and Conditions*: (**c**) CH_2_O, CH_3_COOH:CF_3_CCOOH = 4:1 rt or CH_2_O, SiO_2_/PPA.

**Table 1:** Synthesis of amines **3**.

3	R_1_		Yield, %	M.p., °C
**a**	H	H	42	47–49
**b**	OCH_3_	OCH_3_	82	oil

**Table 2:** Synthesis of amides **4**.

4	R	R_1_	R_2_	Yield, %	M.p., °C
**a**	H	H	COCH_3_	80	85–86
**b**	H	H	SO_2_CH_3_	98	138–139
**c**	H	H	COPhPh	94	192–193
**d**	OCH_3_	OCH_3_	COCH_3_	91	126–128
**e**	OCH_3_	OCH_3_	SO_2_CH_3_	90	49–52
**f**	OCH_3_	OCH_3_	COPhPh	97	200–202

**Table 3:** Comparative yields of the compounds **5** synthesized through two conventional methods.

5	R	R_1_	R_2_	Yield, % (CH_3_COOH:CF_3_COOH = 4:1)	Yield, % (SiO_2_/PPA)
**a**	H	H	COCH_3_	90	94
**b**	H	H	SO_2_CH_3_	80	91
**c**	H	H	COPhPh	84	96
**d**	OCH_3_	OCH_3_	COCH_3_	85	96
**e**	OCH_3_	OCH_3_	SO_2_CH_3_	87	97
**f**	OCH_3_	OCH_3_	COPhPh	87	98

**Table 4:** Synthesis of amides **6** and 4-methyl-1,2,3,4-tetrahydroisoquinolines **7**.

**6**	**R_2_**	**Yield, %**	**M.p., °C**
**a**	COCH_3_	98	Oil
**b**	SO_2_CH_3_	98	Oil
**c**	COPhPh	98	156–157
**7**	**R_2_**	**Yield, %**	**m.p., °C**
**a**	COCH_3_	89	Oil
**b**	SO_2_CH_3_	93	66–67
**c**	COPhPh	96	Oil
